# *Vax1/2* Genes Counteract *Mitf*-Induced Respecification of the Retinal Pigment Epithelium

**DOI:** 10.1371/journal.pone.0059247

**Published:** 2013-03-15

**Authors:** Jingxing Ou, Kapil Bharti, Alessandro Nodari, Stefano Bertuzzi, Heinz Arnheiter

**Affiliations:** Mammalian Development Section, National Institute of Neurological Disorders and Stroke, National Institutes of Health, Bethesda, Maryland, United States of America; Center for Regenerative Therapies Dresden, Germany

## Abstract

During vertebrate eye development, the transcription factor MITF acts to promote the development of the retinal pigment epithelium (RPE). In embryos with *Mitf* mutations, the future RPE hyperproliferates and is respecified as retinal tissue but only in a small portion of the dorsal RPE. Using a series of genetic crosses, we show that this spatial restriction of RPE respecification is brought about by persistent expression of the anti-retinogenic ventral homeodomain gene *Vax2* in the dorso-proximal and both *Vax1* and *Vax2* in the ventral RPE. We further show that dorso-proximal RPE respecification in *Vax2/Mitf* double mutants and dorso-proximal and ventral RPE respecification in *Vax1/2/Mitf* triple mutants result from increased FGF/MAP kinase signaling. In none of the mutants, however, does the distal RPE show signs of hyperproliferation or respecification, likely due to local JAGGED1/NOTCH signaling. Expression studies and optic vesicle culture experiments also suggest a role for NOTCH signaling within the mutant dorsal RPE domains, where ectopic JAGGED1 expression may partially counteract the effects of FGF/ERK1/2 signaling on RPE respecification. The results indicate the presence of complex interplays between distinct transcription factors and signaling molecules during eye development and show how RPE phenotypes associated with mutations in one gene are modulated by expression changes in other genes.

## Introduction

An ideal model to study domain specification during vertebrate central nervous system development is provided by the development of the eye. The eye’s neuroepithelial parts develop from a portion of the rostral neuroectoderm, the optic neuroepithelium, that forms the optic vesicle (OV) and becomes divided into the future retina, the retinal pigment epithelium (RPE), and the optic stalk (OS). While it is known that these domain specifications involve both cell-extrinsic and cell-intrinsic mechanisms (for reviews see [1], [2], [3], many of the molecular details still need to be determined. For example, the *microphthalmia*-associated transcription factor (MITF) regulates RPE specification and development but its integration into functional circuits is only partially understood [4]. In the mouse, *Mitf* is first expressed in the whole OV and then downregulated in the future neuroretinal domain by the visual system homeobox 2 (VSX2) protein [5], [6], [7], leaving it expressed in the presumptive RPE. Loss-of-function mutations in *Mitf* result in loss of pigmentation of the entire RPE combined with hyperproliferation and respecification of a small subdomain of the dorsal RPE as neuroretina [7], [8]. This suggests either that only the dorsal domain of the mutant RPE is exposed to retinogenic inducers that are strong enough to promote retinal development in the absence of *Mitf*, or that the remainder of the *Mitf* mutant RPE is subject to compensatory mechanisms that prevent full RPE-to-retina transitions.

Recent results have indicated that in mice with combined mutations in the transcription factors *Coup-Tf1* and *Coup-Tf2*, the dorsal RPE and OS adopt a neuro-retinal fate and that this fate change is associated with a substantial reduction in the expression of the anti-retinogenic ventral homeodomain gene *Vax1* in the dorsal OS and of *Mitf* in the dorsal RPE [9]. At early stages of development, *Vax1* and its paralog *Vax2* are first partially co-expressed, with *Vax1* showing a gradient from the ventral OS into the ventral retina and *Vax2* an inverted gradient from the ventral retina to the ventral optic stalk [10], [11], [12]. At later stages, the expression domains of *Vax1* and *Vax2* become segregated, with *Vax1* predominantly found in the ventral OS and *Vax2* predominantly found in the ventral retina [10], [11], [12]. The initial co-expression of *Vax1* and *Vax2* may explain why in *Vax1*/*2* double mutants, ventral OS and retina develop as a hyperproliferating *Pax6*-positive retina-like domain and RPE development remains confined to the dorsal domain and in fact expands into the dorsal OS [13]. It was conceivable, therefore, that in *Mitf* mutants, compensatory upregulation of *Vax1* and/or *Vax2* are involved in the territorial limitation of RPE respecification as retina.

These observations prompted us to explore the role of *Vax* genes in *Mitf* mutant backgrounds. We find that in wild type, neither *Vax1* nor *Vax2* are normally present in the dorsal RPE, while in *Mitf* mutants, *Vax2* (though not *Vax1*) is present in the future dorso-proximal RPE. Moreover, at time points when *Vax1* and *Vax 2* are segregated into ventral OS and retina in wild type, both *Vax1* and *Vax2* are retained in the presumptive ventral RPE domain in *Mitf* mutants. Furthermore, while individual *Vax* mutations do not grossly change *Mitf* expression in the presumptive RPE domains, *Vax1/2* double mutants show ventral loss of *Mitf* expression and expansion of *Mitf* expression into the dorsal OS. Intriguingly, *Vax1/2/Mitf* triple mutants that retain at least one functional *Vax* gene copy show hyperproliferation of both dorsal and ventral RPE. In all these mutants, however, the future ciliary margin RPE remains intact, possibly brought about by JAGGED1-NOTCH signaling. The results underscore the complex interplay between distinct transcription factors and signaling molecules and highlight specific mechanisms by which mutations in individual genes can lead to compensatory gene expression changes that finally determine the phenotypic outcomes of these mutations.

## Materials and Methods

### Ethics Statement

All animal experimentations were approved by the NINDS/NIDCD animal care and use committee.

### Mice

The *Vax* knock out alleles, *Vax1^tm1Grl^* and *Vax2^tm1Grl^*, here referred to as *Vax1^−^* and *Vax2^−^*, have been described [14], [15]. They were kept on a mixed C57BL/6;129S1/Sv background. The *Mitf* alleles *Mitf^mi-vga9^* [4] and *Mitf^mi-ew^* [16], both functional null alleles and here referred to as *Mitf ^−^*, were kept on a C57BL/6 background.

### Immunofluoresence, Immunohistochemistry, in situ Hybridization, and RT-PCR

All histological analyses were performed according to previously published protocols [8], [17], [18]. For antigen retrieval, embryo sections were boiled in a microwave oven for 3 minutes in Tris-EDTA (pH 8.5). RT-PCR of dissected RPE and retina were done as described [8]. Antibodies, probes and primer sequences are shown in Table S1.

### Optic Vesicle/Optic Cup Cultures

Optic primordia were obtained from wild-type and mutant embryos and cultured as described [7], as were bead implantations and western blots [7], [8], [19].

### Whole Embryo Cultures

E10−10.5 wild-type embryos were prepared and cultured for 36 hours as described for optic vesicle/optic cup cultures, except that they were floating in the medium with the placenta attached though the amniotic membrane removed, and that DMSO as control or 10 µM γ-secretase inhibitor I (565750, EMD Millipore, USA) was added directly to the culture medium.

### BrdU Incorporation

Pregnant mice were injected intraperitoneally with 100 µg of 5-bromo-2′-deoxyuridine (BrdU; Sigma, St. Louis) in phosphate buffer per gram of body weight. Mice were sacrificed and embryos collected at the indicated times.

## Results

### RPE-to-retina Transition in *Mitf* Mutants is Enhanced by Mutations in *Vax1/Vax2*

**Figure 1:**
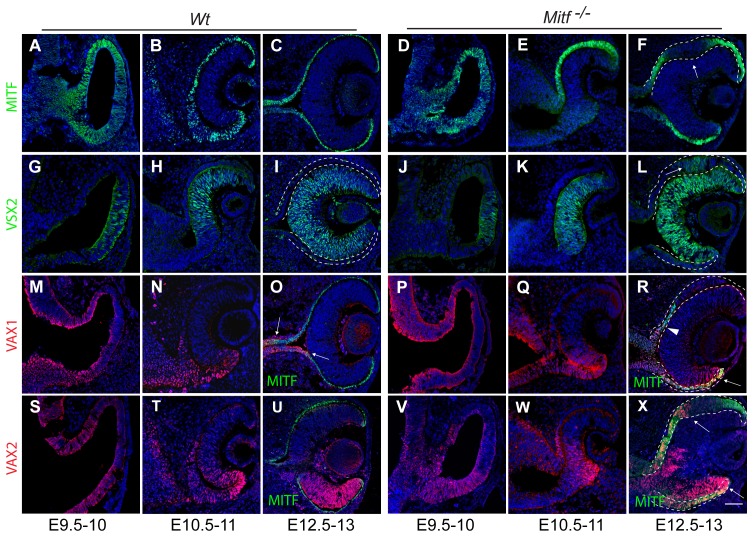
Expression patterns of MITF, VSX2, VAX1 and VAX2 in wild-type and *Mitf* mutant optic vesicles and cups. Embryos of the indicated genotypes were harvested at the indicated times, cryosectioned, and labeled for the indicated proteins. Dorsal is up, and ventral down. The dotted lines mark presumptive RPE. (**A–F**) In both wild type (**A–C**) and mutants expressing non-functional MITF protein (**D–F**), optic vesicles initially show pan-vesicular MITF expression that in optic cups is extinguished in the presumptive retina and so becomes restricted to the presumptive RPE. Note that in mutants, MITF is downregulated in a portion of the dorsal RPE at E12.5 (**F**, arrow). *Mitf* downregulation in the retina is due to complimentary retinal expression of VSX2 (**G–L**). Note that the area of dorsal RPE thickening in mutants also expresses VSX2 (arrow in **L**). (**M–R**) VAX1 protein, present in wild type in presumptive ventral RPE at early stages (**M,N**) but absent later on (**O**, arrows) remains present in ventral RPE in mutant (**R**, arrow). Dorsally, VAX1 expression barely extends into the RPE (**R**, arrowhead). (**S–X**) VAX2 shows prominent ventral retina expression in wild type and mutant at E12.5 (**U**,**X**). In addition, it extends into both the dorsal as well as the ventral RPE in mutant (**X**, arrows). Single channel images of (R) and (X) are provided in Figure S1. Scale bar: 60 µm.

The studies described in this paper involved wild-type mice; mice carrying targeted alleles of *Vax1* or *Vax2*, designated as *Vax1^−^* or *Vax2^−^*; and mice carrying either *Mitf^mi-ew^*, an allele expressing non-functional MITF protein, or *Mitf^mi-vga9^*, a transgenic insertional null allele lacking MITF protein expression. As the latter two alleles are functionally equivalent with respect to developmental eye defects, we designate them here as *Mitf ^−^*. Figure 1 shows the expression patterns of the RPE protein MITF, the retinal protein VSX2 (visual system homeobox protein-2, formerly called CHX10) and VAX1 and VAX2 in wild-type and *Mitf* mutant embryos. In wild type, MITF and VSX2 expression at embryonic day (E) 9.5−12.5 was as previously described (Figure 1A–C,G-I) [7], [20]. Furthermore, as expected from previous in situ hybridizations [10], [11], [12], VAX1 and VAX2 protein were overlappingly expressed in the dorsal and ventral OV at E9.5 (Figure 1M,S) and in the ventral retina and RPE at E10.5 (Figure 1N,T). At later stages, however, VAX1 is only found in the optic stalk (Figure 1O; Figure S1A,B; arrows show the expression boundary between VAX1 and MITF) and VAX2 mostly in the ventral retina (Figure 1U; Figure S1F). Notably, no VAX proteins were found in dorsal or ventral RPE at this stage (Figure S1B,F). Before E10.5, *Mitf ^−/−^* embryos showed no hyperproliferation of the dorsal RPE (compare Figure 1A with D) but such hyperproliferation became gradually apparent thereafter and resulted at E12.5 in a pronounced epithelial thickening in a small portion of the dorsal RPE, concomitant with downregulation of the (non-functional) MITF protein (Figure 1F, arrow; Figure S1C,G). This RPE portion expressed the retinal marker VSX2 (Figure 1L, arrow) and eventually developed as a laminated second retina as previously described [8], [19]. Furthermore, in such mutants, VAX1 was more prominent in the ventral RPE at E10.5 (Figure 1Q) where it stayed on at E12.5 (arrow in Figure 1R; arrowhead marks the VAX1 expression boundary in the dorso-proximal RPE; and Figure S1D), and VAX2 was more prominent in the dorso-proximal RPE at E10.5 (Figure 1W) and in both the dorso-proximal and all of the ventral RPE at E12.5 (arrows in Figure 1X and Figure S1H).

**Figure 2:**
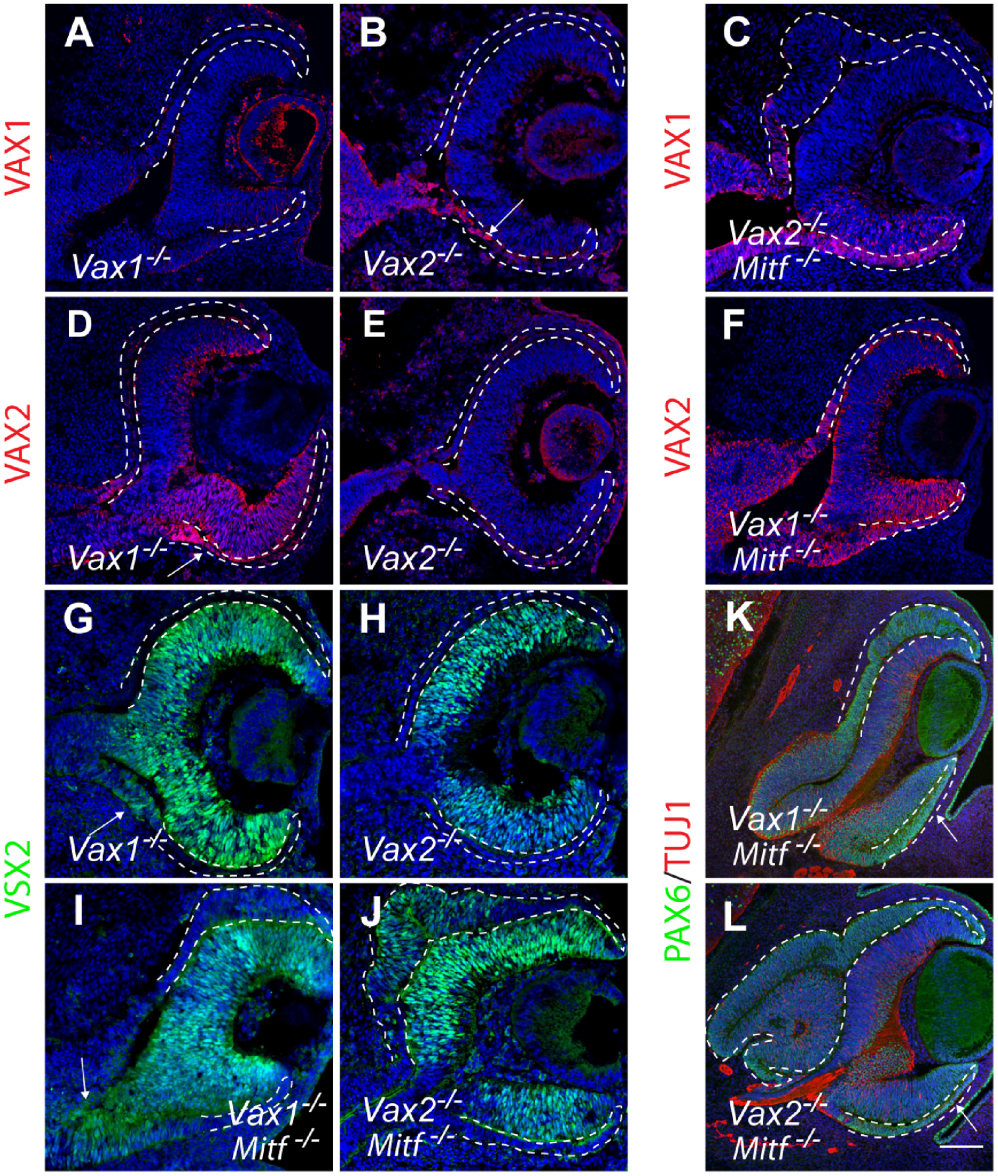
*Vax* mutations exacerbate the dorsal RPE phenotypes in *Mitf ^−/−^* optic cups. Coronal sections are from E12.5 embryos (**A–F**; **G–J**) and E14.5 embryos (**K**,**L**). (**A**,**B**,**D**,**E**) In *Vax1* ^−/−^ mutants, VAX2 extends into the ventral RPE (**D**, arrow), and in *Vax2^−/−^* mutants, VAX1 extends into the ventral RPE (**B**, arrow). Absence of labeling on control sections (*Vax1^−/−^* labeled for VAX1, **A**, or *Vax2^−/−^* labeled for VAX2, **E**) indicates antibody-specificity. (**C**,**F**) *Vax2^−/−^;Mitf ^−/−^* embryos show massive dorsal RPE hyperproliferation in areas negative for VAX1, but *Vax1^−/−^;Mitf ^−/−^* embryos show little dorsal RPE hyperproliferation. (**G,H,I,J**) Corresponding sections labeled for VSX2. Note that VSX2 expression is absent in the dorsal RPE of *Vax1^−/−^* and barely visible in the dorsal RPE of *Vax1^−/−^;Mitf ^−/−^* mutants (**G,I**), but present in the hyperproliferating dorsal RPE region of *Vax2^−/−^;Mitf ^−/−^* mutants (**J**). Also note VSX2 expression at the ventro-proximal OS/RPE boundary in **G,I** (arrows) but absence of VSX2 expression in the ventral RPE. (**K,L**) Milder dorsal RPE thickening in E14.5 *Vax1^−/−^;Mitf ^−/−^* mutants (**K**) compared to *Vax2^−/−^;Mitf ^−/−^* mutants (**L**). Note that in both **K,L**, the ventral RPE remains largely unchanged at this stage (arrow). Scale bar: 60 µm (**A–J**); 130 µm (**K**,**L**).

To directly test the possibility that retention of VAX proteins in the dorso-proximal and ventral RPE counteracts the phenotypic effects of *Mitf* mutations, we generated *Vax1/Mitf* and *Vax2/Mitf* compound mutants and compared them with the respective single mutants. In *Vax1* or *Vax2* single mutants, *Mitf* expression was not grossly changed in the presumptive RPE domains (Figure S1I,J), but VAX1 was retained in the ventro-proximal RPE of E12.5 *Vax2* mutants (arrow in Figure 2B), and VAX2 was retained in the ventro-proximal RPE of E12.5 *Vax1* mutants (arrow in Figure 2D). Nevertheless, the expression of PAX6 and PAX2, known to reciprocally repress each other’s functions to define the OS/optic cup boundary [21], was unchanged in all single mutants tested (Figure S1K–R). In the compound mutants, dorsal RPE thickening at E12.5 was much more pronounced in *Vax2/Mitf* mutants (Figure 2C) compared to *Vax1/Mitf* mutants (Figure 2F) or *Mitf* single mutants (see Figure 1F). This result likely reflects the fact that in *Mitf* mutants, VAX2 expression was retained in the dorso-proximal RPE (Figure 1X and Figure S1H) while VAX1 expression was not (Figure 1R and Figure S1D). The more pronounced dorsal RPE thickening in *Vax2/Mitf* double mutants was also reflected by a more pronounced expression of VSX2 in this area (compare Figure 2I with J). The difference between the two compound mutants became even bigger at E14.5, when the dorsal RPE of *Vax2/Mitf* mutants was massively expanded by comparison with that of *Vax1/Mitf* mutants (Figure 2K,L). The compound mutant RPEs also retained strong PAX6 expression (Figure 2K,L) while in wild-type RPE, PAX6 was gradually lost [19].

**Figure 3:**
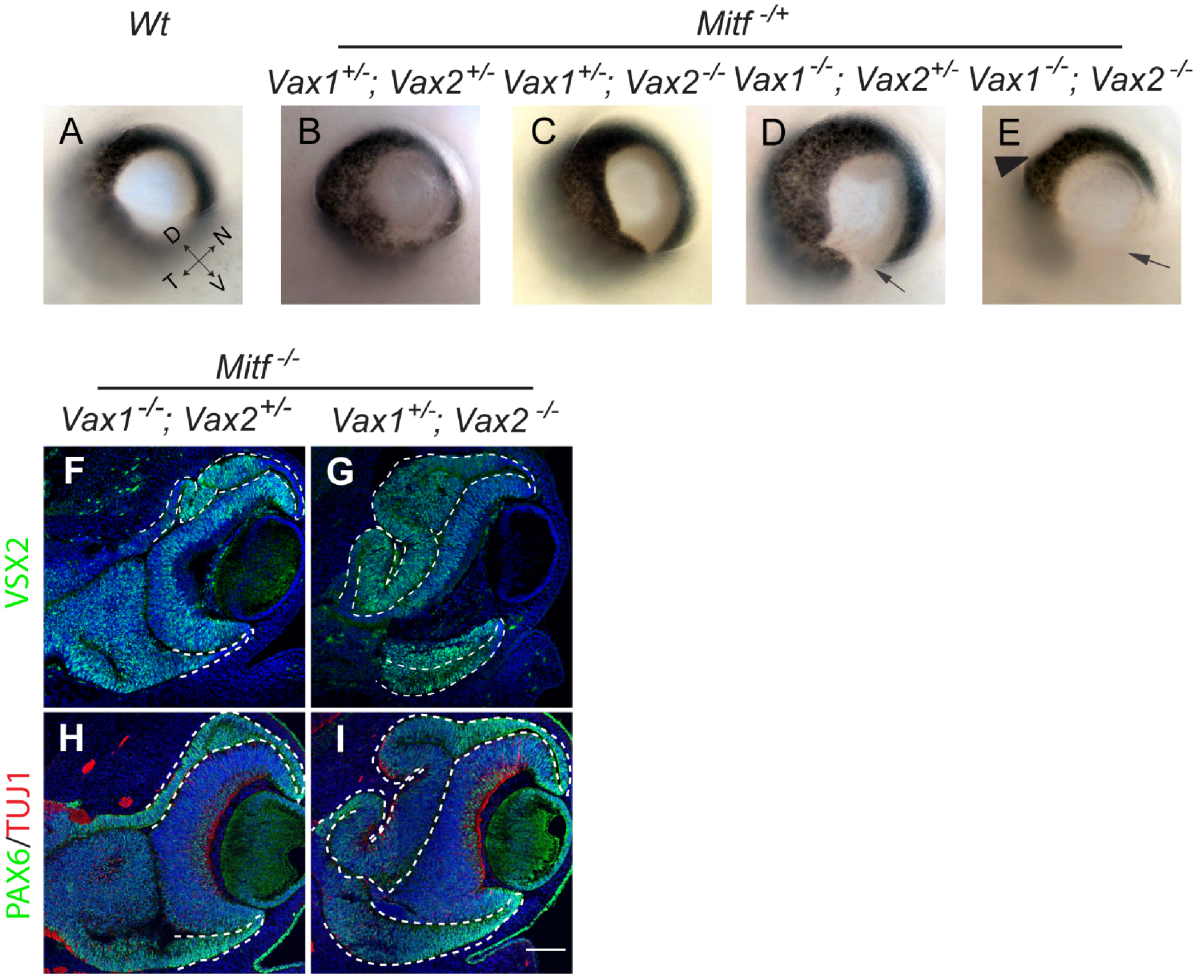
*Vax1* and *Vax2* redundantly limit retinogenesis in the presumptive dorso-proximal and ventral RPE domains of the *Mitf* mutant optic cups. (**A–E**) In E14.5 *Mitf^ +/−^* heterozygotes, RPE defects are *Vax1* and *Vax2* gene dose-dependent. In the total absence of Mitf (**F–I**), VSX2 and PAX6 expression are seen in the ventral RPE regardless of whether only one copy of *Vax2* (**F**,**H**) or one copy of *Vax1* (**G**,**I**) is present. Also note that dorsal RPE thickening and VSX2 and PAX6 expression are more prominent when VAX2 is totally missing (**G,I**) as opposed to when VAX1 is totally missing (**F,H**). Scale bar: 200 µm (**A–E**); 130 µm (**F–I**). Coordinates in (**A**): D – dorsal; V – ventral; T – temporal; N – nasal.

Interestingly, in none of the above compound mutants was there any thickening or VSX2 expression in the corresponding ventral RPE. This was conceivably due to the fact that in this domain, both VAX1 and VAX2 were overlappingly retained in *Mitf* mutants (see Figure 1Q,R,W,X and Figure S1D,H) and that either protein might compensate for the lack of the other. In fact, it has been observed previously that *Vax1/Vax2* compound mutants show a massive thickening of the ventral optic neuroepithelium, with most cells positive for PAX6 and negative for PAX2, and none of them expressing the RPE marker DCT [13]. Consistent with these results, we find that although the dorsal RPE/OS of *Vax1^−/−^;Vax2^−/−^;Mitf ^+/+^* mutants showed strong MITF expression, MITF expression was absent in the hyperproliferating ventral RPE/OS of such mutants or, interestingly, both dorsal and ventral RPE/OS in *Vax1^−/−^;Vax2^−/−^;Mitf ^+/−^* mutants except at their distal margins (Figure S2A–C). We, therefore, reasoned that *Vax1/Vax2/Mitf* triple homozygotes might not show a phenotype in the ventral RPE beyond that of *Vax1/2* double homozygotes. Hence, in order to test for *Vax1*/*Vax2* redundancies in this domain, we left at least one copy of a *Vax* gene intact. Direct inspection of E14.5 eyes showed that as long as at least one copy of wild-type *Mitf* was retained, the presence of one copy of *Vax1* (and none of *Vax2*) led to near normal ventral pigmentation (Figure 3C), the presence of one copy of *Vax2* (and none of *Vax1*) only to a minor gap in ventral pigmentation (arrow in Figure 3D), and the absence of both *Vax1* and *Vax2* to loss of RPE pigmentation in the ventral eye as previously described for *Vax1^−/−^;Vax2^−/−^;Mitf ^+/+^* mutants [13] (arrow in Figure 3E), and also in the dorsal proximal part of the eye (arrowhead in Figure 3E). In contrast, as shown in Figure 3F–I, in the total absence of functional *Mitf*, the presence of one copy of *Vax2* (and none of *Vax1,* Figure 3F) or one copy of *Vax1* (and none of *Vax2*, Figure 3G) led to ventral RPE thickening and increased VSX2 and PAX6 expression compared with the respective *Vax1/Mitf* or *Vax2/Mitf* compound mutants (see Figure 2). The triple mutants also showed a more pronounced dorsal RPE thickening and VSX2 expression compared with the respective *Vax1/Mitf* and *Vax2/Mitf* compound mutants. These results, summarized in Table 1, suggest that in the ventral RPE of *Mitf* mutants, *Vax1* and *Vax2* are indeed partially redundant for reducing retina transitions, while in the dorso-proximal RPE of *Mitf* mutants, *Vax2* alone limits such retina transitions.

**Table 1:** RPE phenotypes in *Vax1/Vax2/Mitf* mutants.

Genotypes	RPE phenotypes (Dorsal: D; Ventral: V; Proximal: P)					
	Thickening/respecification		VSX2 expression		Pigmentation	Emergence age
*Mitf ^−/−^*	D: +	V: −	D: +	V: −	Fully lost	E12−12.5
*Vax1^−/−^*	D: −	V: − *	D: −	V: − *	V: coloboma	
*Vax2^−/−^*	D: −	V: −	D: −	V: −	V: mild coloboma	
*Vax1^−/−^;Mitf ^−/−^*	D: +/− **	V: −	D: +/− **	V: −	Fully lost	E12.5
*Vax2^−/−^;Mitf ^−/−^*	D: +++	V: −	D: +++	V: −	Fully lost	E10.5
*Vax1^−/−^;Vax2^+/−^;Mitf ^−/−^*	D: ++	V: +	D: ++	V: +	Fully lost	E11.5
*Vax1^+/−^;Vax2^−/−^;Mitf ^−/−^*	D: ++++	V: ++	D: ++++	V: ++	Fully lost	E10.5
*Vax1^+/−^;Vax2^+/−^;Mitf ^+/−^*	D: −	V: −	D: −	V: −	Overall less pigmented	
*Vax1^−/−^;Vax2^+/−^;Mitf^ +/−^*	D: −	V: −	D: −	V: −	V: coloboma	
*Vax1^+/−^;Vax2^−/−^;Mitf ^+/−^*	D-P: +/− ***	V: −	D-P: +/− ***	V: −	D-P: +/− ***; V: mildcoloboma	E12.5
*Vax1^−/−^; Vax2^−/−^;* *Mitf ^+/−^*	D-P: +++	V-P: +	D-P: +++	V-P: +	D-P: −;V: severe coloboma	E10.5

* In all mutants that are *Vax1^−/−^*, the prospective ventral RPE domain is present in the early optic vesicle/optic cup stage, but gradually displaced by the overgrowing presumptive ventral optic stalk domain that also abnormally contains VSX2-expressing cells, resulting in ventral coloboma after E14;

** Interestingly, about 50% of the *Vax1^−/−^;Mitf ^−/−^* embryonic eye sections showed only mild dorsal RPE phenotypes (see Figure 2F). It is possible that loss of VAX1 functions increases the local dosages of antiretinogenic factors such as VAX2, JAGGED1, or TFEC [19] but such changes may be too subtle to be detected by immunostaining or *in situ* hybridization.

*** Although the *Vax1^+/−^;Vax2^−/−^;Mitf ^+/−^* embryos appeared to have largely normal RPE pigmentation (Figure 3C), on sections there were some patches of thickened dorsal-proximal RPE subdomains that express VSX2 at very low levels.

### The Role of FGF in *Mitf* Mutant RPE Development

The above observations suggest that the RPE- and OS-to-retina respecifications are associated with increased cell proliferation. In fact, BrdU incorporation assays showed increased DNA synthesis in E10.5 *Mitf ^−/−^* RPE compared to the corresponding wild-type RPEs (Figure S3A–E). Earlier results also showed that MITF has prominent antiproliferative activities [22], [23], [24] and that FGF signaling reduces *Mitf* expression in the RPE and enhances cell proliferation [7], [25]. In addition, feedback loops may allow MITF to regulate the very signaling pathways that influence its own activities [26], [27], and so we tested whether *Mitf* and *Vax1/2* mutations might exert their effects at least in part through changes in FGF signaling.

**Figure 4:**
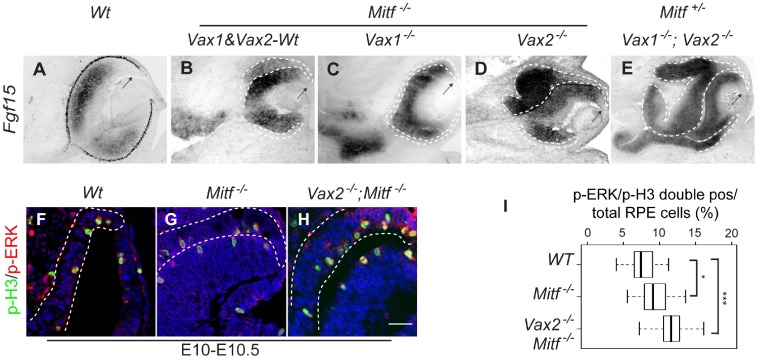
FGF-MAP kinase signaling regulates RPE-to-retina transition in *Mitf* mutants. (**A–E**) In situ hybridization for *Fgf15*. In wild type (**A**), *Fgf15* is normally restricted to the neural retina but is absent in the distal retina (arrow). (**B–E**) Ectopic expression of *Fgf15* in the dorsal RPE is seen in *Mit ^−/−^* (**B**), *Vax2^−/−^;Mitf ^−/−^* (**D**), and *Vax1^−/−^;Vax2^−/−^;Mitf ^+/−^* (**E**) though not in *Vax1^−/−^;Mitf ^−/−^* mutants (**C**). Note that as in wild type, the dorsal future ciliary margin shows little *Fgf15* labeling (arrow in **B–E**). (**F–H**) Increased numbers of p-H3/p-ERK double-positive cells in the RPE of E10−10.5 *Mitf ^−/−^* (**G**) and *Vax2^−/−^;Mitf ^−/−^* mutant (**H**) as compared to wild-type RPE (**F**). (**I**) Quantitation of the results obtained from sections as in **F–G**. Box plots show minimal, 25^th^ percentile, median, 75^th^ percentile and maximal percentages. Significance determined by Student’s *t-*test: *: p<0.05; ***: p<0.001 (2–3 sections per embryo, 6–10 embryos per genotype). Scale bar: 150 µm (**A–E**), 25 µm (**F–H**).

To test for the role of FGF signaling, we focused on FGF15. This factor is the major FGF expressed in the embryonic mouse retina but is absent in the RPE [19]. Nevertheless, RNA for its cognate receptors, FGFR1 and FGFR2, were found both in retina and RPE (Figure S3F). By in situ hybridization, *Fgf15* was ectopically expressed in the dorsal RPE of *Mitf ^−/−^* single mutants and even more prominently in the massively expanded RPE of *Vax2^−/−^*;*Mitf ^−/−^* double and *Vax1^−/−^;Vax2^−/−^;Mitf ^+/−^* triple mutants (Figure 4B,D,E), though only mildly in approximately half of *Vax1^−/−^*;*Mitf ^−/−^* double mutants (Figure 4C). Nevertheless, high levels of FGF15 transcripts were seen in the abnormally thickened OS domains of both *Vax1^−/−^*;*Mitf ^−/−^* (Figure 4C) and *Vax1^−/−^;Vax2^−/−^;Mitf ^+/−^* mutants (Figure 4E). As expected from previous results studying the role of FGF1 and FGF2 [28], [29], [30], [31], [32], increased expression of FGF15 led to increased staining for activated extracellular signal-regulated kinases 1/2 (ERK1/2) [17], [18], [33] in the dorsal RPE and correspondingly higher numbers of mitotic cells as evidenced by increased labeling for phospho-histone H3 (p-H3)-positivity (Figure 4F–I). We then confirmed the correlation between increased ERK1/2 signaling and RPE-to-retina transitions by applying the MEK inhibitor PD 98095 (MEKi) on heparing-acrylic beads to optic vesicle explant cultures as described previously [7]. As shown in Figure S3G, inhibition of ERK1/2 signaling in wild-type cultures reduced the levels of pERK, p-H3, and Cyclin-D, regardless of whether extra amounts of FGF were added or not. In fact, in similar experiments performed with *Mitf ^−/−^* (Figure S3H–M) and *Vax2^−/−^*;*Mitf ^−/−^* optic vesicle cultures (Figure S3N–P), inhibition of ERK1/2 signaling led to reduced numbers of p-H3-positive RPE cells and reduced expression of VSX2. Taken together, these results suggest that MITF and VAX proteins regulate FGF expression in the developing RPE and that in turn, as expected, FGF-ERK1/2 signaling then leads to increased cell proliferation and domain respecification.

### The Role of NOTCH Signaling in *Mitf* Mutant RPE Development

**Figure 5:**
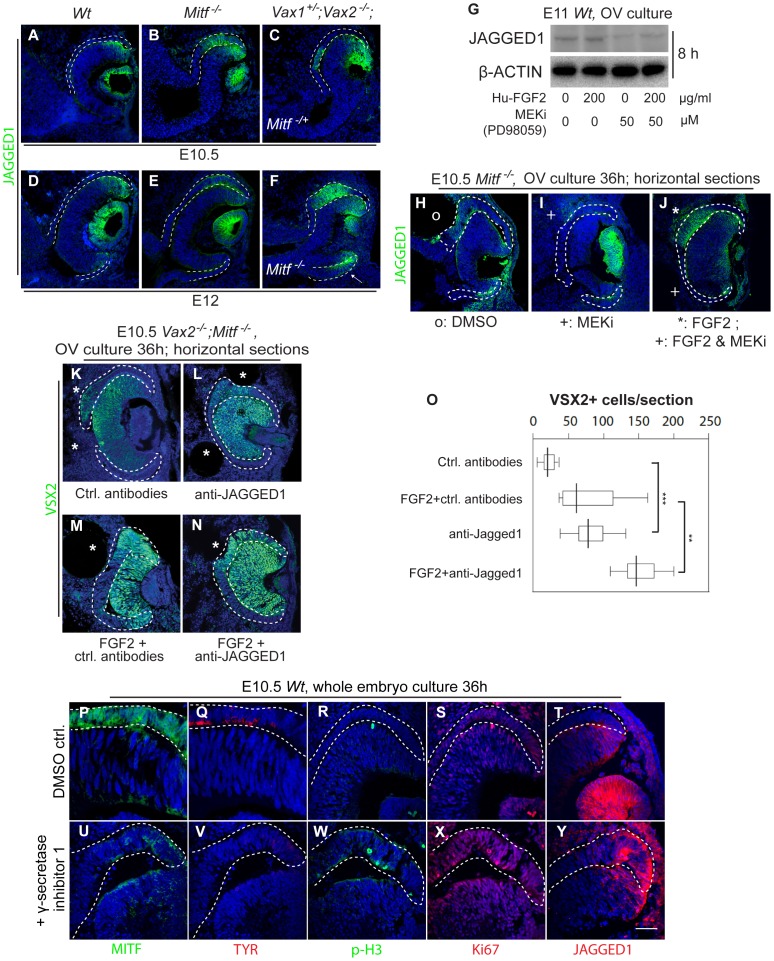
JAGGED1-NOTCH regulates RPE proliferation and specification. (**A–F**) Optic cups of the indicated genotypes were harvested at the indicated times and stained for JAGGED1. Note that in wild type and all indicated mutants, JAGGED1 is expressed in the dorsal distal retina and in (**F**) also in the E12.0 ventral distal retina and RPE (arrow), in addition to its prominent expression in the lens vesicle. Also note that a dorsal RPE subdomain in *Mitf* single or *Vax1/2/Mitf* triple mutants expresses JAGGED1 at both E10.5 and E12.0. (**G**) Western blots for the indicated proteins from wild-type OV cultures eight hours after exposure to human FGF2 and/or MEK1/2 inhibitor (MEKi). Note decrease in the level of JAGGED1 in the presence of MEKi. (**H–J**) JAGGED1 expression in sections of E10.5 *Mitf ^−/−^* optic cups 36 hours after exposure to control beads (**H**, marked by o), MEKi bead (**I**, marked by +) or after co-implantation of an FGF2 bead (**J**, marked by *) and a bead coated with both FGF2 and MEKi (**J**, marked by +). Note that these are horizontal sections and that FGF2 and FGF2+MEKi have differential effects within the same OV culture. (**K–N**) Effects of antibody neutralization of JAGGED1 on VSX2 expression in *Vax2^−/−^*;*Mitf ^−/−^* cultures. Compared to control antibodies (**K**), anti-JAGGED1 antibodies modestly increased VSX2 expression in the RPE (**L**) and further increased it when the beads were double-coated with FGF2 (**N**). Note that the anti-JAGGED1 effect in (**L**) is seen only in a subdomain of the RPE, consistent with the fact that JAGGED1 expression was confined to the thickening portion of the *Mitf* single and *Vax/Mitf* compound mutant RPE. (**O**) Quantitation of VSX2-positive cells in optic cup cultures. Box plots show minimal, 25^th^ percentile, median, 75^th^ percentile and maximal percentages of VSX2-positive cells in the RPE per section (one section per embryo, and 8–9 embryos for each treatment). Significance determined by Student’s *t-*test: p<0.01 for control versus FGF2, and FGF2 versus FGF2+anti-JAGGED1; p<0.001 for control versus anti-JAGGED1, anti-JAGGED1 versus FGF2+anti-JAGGED1. (**P–Y**) Wild-type whole embryo cultures after 36-hour exposure to DMSO or NOTCH antagonist γ-secretase inhibitor 1. Note that under control conditions, RPE markers MITF (**P**) and TYROSINASE (TYR)(**Q**) were normally expressed, the numbers of phosphorylated histone H3 (p-H3) positive (**R**) and Ki67 positive (**S**) cells in the RPE were low, and JAGGED1 expression (**T**) was normal in the distal future retina. After γ-secretase 1 incubation, however, MITF and TYR expression was greatly reduced, the number of proliferative cells was elevated, and the expression territory of JAGGED1 expanded into the distal RPE (**U–Y**). Scale bar: 80 µm (**A–F**), 60 µm (**H–N**), 25 µm (**P–Y**).

The above results showed a role for *Vax* genes to limit RPE-to-retina transition in the proximal portion of the RPE but did not address why RPE-to-retina transitions were also limited towards the distal RPE domain. In fact, in all single and double mutants and even the *Vax1/Vax2/Mitf* triple mutants, the distal RPE, which later contributes to ciliary body and iris, lacked expression of retinal markers, including VSX2 (for instance, Figures 1L; 2J; 3F,G and S4B,C,E,F), SOX2 (Figure S4B,C,E,F), and FGF15 (Figure 4A–E, arrows), and retained MITF expression and its monolayer characteristics (for instance Figures 1F,X; and Figure S2). Interestingly, at the OV stage, *Jagged1*, encoding a NOTCH ligand, is expressed in the lens placode and the dorsal future retina and then stays on in lens and distal retina ([34], [35]; and Figure 5A,D for JAGGED1 protein expression), and the gene encoding one of its receptors, NOTCH2, is expressed in the RPE including its distal tip [34], [35]. By comparison, *Dll1*, the gene encoding the NOTCH ligand DLL1, is initially expressed in the optic stalk and, once an optic cup is formed, expands into the proximal retina [34], [35]). Interestingly, *JAGGED1* heterozygous mutations in humans cause anterior eye defects [36] as do mutations in the homologous gene in mice [37], [38]. It was conceivable, therefore, that in the mammalian optic cup, JAGGED1 might be one of the factors that limits retinal development in the distal RPE of *Mitf* and *Vax/Mitf* mutants.

In *Mitf* single or *Vax/Mitf* compound mutants, which as shown above are prone to RPE hyperproliferation and respecification, JAGGED1 was indeed retained at E10.5 and thereafter in the distal retina. It was, however, also present in the dorsal RPE, even when there was not yet any dorsal thickening at the early optic cup stage in some mutants (E10.5 *Mitf ^−/−^* and *Vax1^+/−^;Vax2^−/−^;Mitf ^−/+^* mutants are shown in Figure 5B,C). In addition, in E12.5 *Vax1/2/Mitf* triple mutants, JAGGED1 was seen in the ventral distal RPE (arrow in Figure 5F, shown for *Vax1^+/−^;Vax2^−/−^;Mitf ^−/−^*). Unlike JAGGED1, however, DLL1 did not show clear ectopic labeling in *Mitf* mutant RPE and so was unlikely to be a major contributor regulating distal RPE development (not shown).

While the presence of JAGGED1 in the distal retina was consistent with its presumed anti-retinogenic function for the NOTCH2-expressing adjacent RPE, its presence in the hyperproliferating RPE of *Mitf* mutants and *Mitf/Vax* compound mutants was intriguing as these RPE domains, unlike the distal retinal domains that develop as ciliary margin/ciliary body, develop as an expanded retina, and, for that matter, the underlying normal retina does not develop as an RPE. Hence, we reasoned that either JAGGED1 activity in the transdifferentiating RPE was counteracted by the massive expression of FGF/MAP kinase signaling described above, or that it was without activity, for instance for lack of an appropriate receptor. The latter, however, was unlikely as neuroretinal tissues express NOTCH3 [35] to which JAGGED1 binds [39]. To test for possible FGF/JAGGED1 interactions, we again resorted to the OV explant culture system. While addition of FGF2 did not markedly alter the levels of JAGGED1 in western blots of wild-type cultures kept for 8 hours, addition of MEKi, with or without additional FGF2, reduced its levels compared to β-actin (Figure 5G). To analyze these effects histologically, we then implanted beads coated with MEKi, FGF2, or FGF2+MEKi into *Mitf* ^−/−^ cultures. The results (Figure 5H–J) indicated locally decreased JAGGED1 expression in the vicinity of MEKi-coated or FGF2/MEKi doubly-coated beads and increased JAGGED1 expression in the vicinity of FGF2-coated beads. These results support the above observation that the dorsal hyperproliferating RPE domain of *Mitf* and *Vax/Mitf* mutants expressed JAGGED1 ligands *in vivo*. To address the question of whether JAGGED1 shows activity in this region, we used neutralizing JAGGED1 antibodies in *Vax2/Mitf* mutant OV cultures and assayed for changes in RPE histology and gene expression. In fact, anti-JAGGED1-coated beads led to a modest increase in VSX2 expression in a confined RPE domain, while beads coated with anti-JAGGED1 and FGF2 further increased the size of, and VSX2 expression, in the putative RPE domain (Figure 5L,N, quantitation of VSX2-positive RPE cells in Figure 5O). The results suggest that JAGGED1, which is expressed within the hyperproliferating RPE, partially counteracts the well-known pro-retinogenic effect of FGFs.

Constitutively active NOTCH signals can enhance RPE cell proliferation and lead to formation of pigmented tumors in the eye [40]. To test whether inhibiting NOTCH signaling would affect normal RPE development, E10.5 wild-type mouse embryos were cultured with or without a NOTCH-blocking compound, γ-secretase inhibitor I. As expected, in the 36-hour control group, the RPE of such cultured embryos showed normal MITF and TYROSINASE (TYR) expression (Figure 5P,Q), very few p-H3 and Ki67-positive cells (Figure 5R,S), and normal JAGGED1 expression in the future ciliary margin retina (Figure 5T & Figure S4G). With NOTCH activities blocked, however, MITF and TYR expression decreased sharply, the number of p-H3 and Ki67-positive cells in the RPE increased, and the JAGGED1-positive territory expanded into the distal RPE (Figure 5U–Y & Figure S4H). It appears, therefore, that NOTCH activity is required to determine the proper RPE/ciliary boundary and maintain normal cell proliferation and differentiation of the RPE. By extension, upregulated JAGGED1 in the dorsal RPE of *Mitf* and *Mitf/Vax* mutants, likely leading to upregulated NOTCH signaling in this domain, may function to partially counteract the strong FGF-ERK1/2 mediated RPE respecification. Nevertheless, it is likely that JAGGED1 acts in conjunction with other signaling and transcriptional mechanisms acting in the corresponding domains.

**Figure 6:**
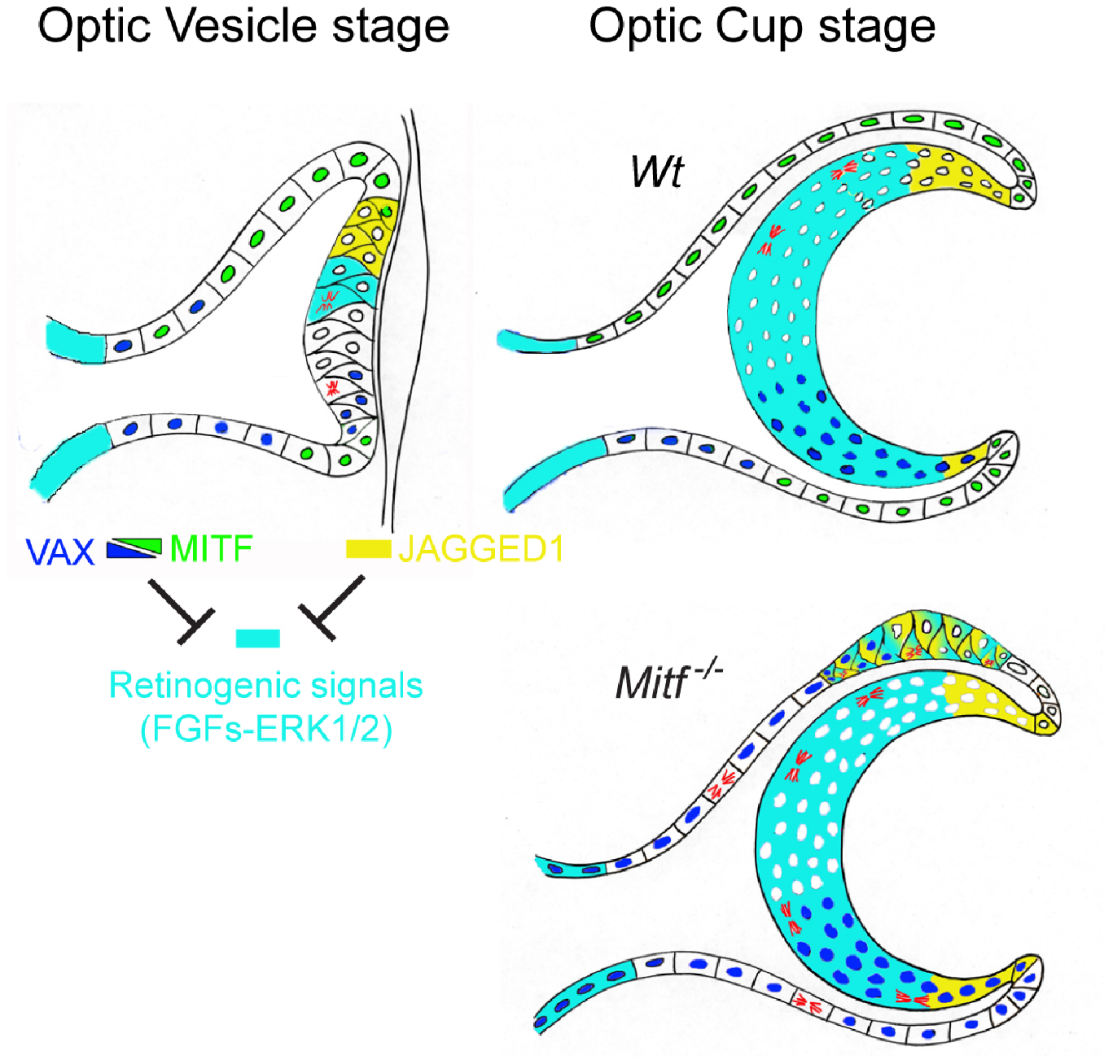
VAX, MITF and JAGGED1-NOTCH counteract FGF-ERK signaling in mediating proper compartmentalization of the optic neuroepithelium. The genetic analyses presented in this paper suggest that at the optic cup stage, the gradients of VAX and MITF protein restrict the future neuroretinal domain to the central distal portion of the optic neuroepithelium. JAGGED1 expression, first dorsal and then ventral, counteract retinogenic signals in the future ciliary margin. Loss of MITF functions, such as due to loss-of-function mutations in its gene, lead to RPE abnormalities including a dorsally restricted RPE-to-retina transition mediated by strong local retinogenic FGF signals. These retinogenic signals are counteracted by the antiretinogenic VAX proteins that remain present in the RPE, helping to dorsally limit RPE respecification.

In sum, our results, summarized in Figure 6, indicate that in the proximal part of both the dorsal and ventral RPE, persistent expression of *Vax* genes are responsible for restricting *Mitf*-associated RPE transdifferentiation. In contrast, in the distal, anterior part, JAGGED/NOTCH signaling may prevail and so help to prevent transdifferentiation even in *Vax1/2/Mitf* triple mutants.

## Discussion

It is well established that mutations of mouse *Mitf* and its orthologs in other species lead to abnormalities in the RPE that include loss of pigmentation, hyperproliferation, and eventual differentiation of a small dorsal subdomain as a second retina instead of RPE [4], [23], [25], [41], [42]. The mechanisms responsible for this dorsally restricted RPE respecification, however, are only partially understood. It has been shown that mutations in *Vsx2* can alleviate [5], and reductions in *Pax6* gene dose greatly exacerbate [19], the dorsal RPE pathology associated with *Mitf* mutations. Here, we investigated whether the ventral homeodomain proteins VAX1 and VAX2, known for their anti-retinogenic role during development [13], might also play a role in RPE respecification in *Mitf* mutants. Both proteins are normally expressed in the future RPE, though only at the OV stage and no longer at the later optic cup stage. Interestingly, we found that at the optic cup stage, *Mitf* mutations lead to an abnormal retention of VAX2 in the dorso-proximal RPE and of both VAX1 and VAX2 in the ventral RPE. Furthermore, introduction of targeted mutations in *Vax2* into *Mitf*-mutant backgrounds allows for a shift of the dorso-proximal boundary of RPE hyperproliferation and VSX2 expression, and hence initiation of the retinal fate, towards the OS. Moreover, when only a single *Vax* gene copy was left intact, the ventral RPE also underwent efficient retinal re-specification. These results suggest that once present in the RPE, VAX1 and VAX2 counteract the FGF/MAP kinase-mediated hyperproliferation and retinal respecification. This interpretation is consistent with the earlier observation that combined early loss of these two proteins leads to giant retinae developing from the ventral optic neuroepithelium and OS [13].

It is conceivable that the effects of *Vax1/2* in the RPE are not strictly due to their specific retention in the *Mitf*-mutant RPE but result from indirect effects of their normal expression in OS and ventral retina. Two observations, however, argue against this possibility. First, when *Vax1*, whose expression does not extend substantially into the dorso-proximal RPE of *Mitf* mutants, is lost, the proximal boundary of the dorsal RPE re-specification remains largely unchanged. In contrast, when *Vax2*, whose expression does extend into the dorso-proximal RPE of *Mitf* mutants, is lost, the proximal boundary of the dorsal RPE hyperproliferation and VSX2 expression zones are shifted proximally. Second, the *Mitf* mutant ventral RPE does not undergo re-specification as long as two copies of either *Vax1* or *Vax2* are present. In other words, it does not matter whether the remaining VAX protein is largely absent from the adjacent ventral retina, as is VAX1, or is present in the adjacent ventral retina, as is VAX2. We believe, in fact, that even the use of Cre-recombinase conditional *Vax* gene mutants might not easily allow for a clear discrimination between RPE-specific and indirect mechanisms as RPE drivers acting early may also affect the future OS and retina, and those acting later in the RPE may eliminate *Vax* genes too late to shift the boundaries of RPE re-specification. In any event, the results show that *Vax* genes can have anti-retinogenic activities not only in the future retina but also in the future RPE.

While the above considerations point to the role of *Vax* genes in helping to set the proximal boundaries of respecification of the *Mitf*-mutant RPE, they do not address the question of what mechanisms are responsible for setting the distal boundary of re-specification. It has previously been seen that many pathways including WNT [43], [44], [45], TGF-β [25] and BMP [46] signaling are persent around the ciliary margin. Here, we focused on NOTCH signaling because we found that the NOTCH ligand JAGGED1 was present in the dorsal distal retina of all mutants analyzed in this study and also in the ventral distal RPE of *Vax1/2/Mitf* mutants. The facts that NOTCH2, a JAGGED1 receptor, is expressed in the adjacent RPE [34], [35], and that inhibition of NOTCH signaling led to abnormal RPE development suggest that NOTCH signaling plays a critical role in keeping the distal RPE from differentiating into retina. More intriguing, however, was the observation that JAGGED1 was also seen in the hyperproliferating *Mitf* mutant dorsal RPE. It was conceivable, therefore, that this mutant RPE domain resembles the distal retinal domain of wild type. Nevertheless, the *Mitf* single or *Vax2/Mitf* double mutant dorsal RPE also expressed SOX2, a gene not normally found in the distal retina, and it expressed FGF15, which is also absent in the distal retina in wild type or any of the single and compound mutants tested in this study. In fact, direct assays for the activity of JAGGED1 using anti-JAGGED1 antibodies in *Mitf* mutant OV explant cultures suggested that JAGGED1 counteracts, at least partially, the *Vax/Mitf/*FGF-mediated RPE hyperproliferation and ectopic VSX2 expression. Hence, expression of *Jagged1* in the mutant RPE may be seen as resulting from induction of a regulatory mechanism to limit RPE hyperproliferation and RPE-to-retina transitions. Interestingly, constitutive activation of NOTCH signaling using activated intracellular NOTCH in the RPE enhances RPE cell proliferation and results in the formation of pigmented tumors via an RBP-Jκ-dependent mechanism [40]. Nevertheless, we have not been able to clearly observe RBP-Jκ induction in the abnormal RPE of *Mitf* mutants and their various genetic combinations.

## Supporting Information

Figure S1 Expression patterns of MITF, PAX6 and PAX2 remain largely unchanged in *Vax1*, *Vax2* and *Mitf* single mutant optic cups. Single channel confocal images of MITF and VAX1, and MITF and VAX2 expression patterns in E12.5 wild type (*Wt*; **A**,**B**,**E**,**F**), and *Mitf ^−/−^* (**C**,**D**,**G**,**H**). Arrows indicate the expression of VAX proteins in the *Wt* optic stalk (**B**) and *Mitf ^−/−^* RPE (**D**,**H**). MITF expression is normal in the RPE of *Vax1^−/−^* (**I**) and *Vax2^−/−^* mutants (**J**). (**K–R**) Normal expression of PAX6 and PAX2 in retina and OS of *Wt* and mutant embryos. Note enhanced PAX6 expression in the dorsal RPE of *Mitf ^−/−^* mutants, confirming previous observations [19]. Scale bar: 80 µm.
(TIF)

Figure S2 MITF expression in *Vax1/Vax2* double mutants. Compared to wild type (**A**), MITF expression is expanded into the dorsal OS in *Vax1/Vax2* double homozygous mutants at E14.5 (**B**). (**C**) Interestingly, *Vax1^−/−^;Vax2^−/−^;Mitf^ +/−^* mutants show dorsal RPE thickening and loss of MITF expression in the dorso-proximal RPE but retention of MITF expression in the distal RPE. Scale bar: 180 µm.
(TIF)

Figure S3 The thickening of *Mitf* mutant RPE is associated with cellular hyperproliferation. BrdU was injected intraperitoneally into pregnant mice, and mice were sacrificed 2 hours thereafter. Embryos were fixed and sectioned coronally. Sections were stained with antibodies against BrdU and double labeled for CYCLIN D1 or Ki67. (**A**, **B**) At E10.5, BrdU/CYCLIN D1 double label in wild-type and *Mitf ^−/−^* eyes. Already at this stage before overt dorsal RPE thickening, *Mitf ^−/−^* RPEs show increased BrdU labeling compared to wild type. (**C**) Quantitation of BrdU positive cells/per total cells in the dorsal RPE subdomain of wild type and *Mitf ^−/−^* embryos. Box plots show minimal, 25^th^ percentile, median, 75^th^ percentile and maximal values of the respective percentages. Significance determined by Student’s *t-*test: ***: p<0.001. For quantitation, 6–10 embryos of each genotype and 2–3 sections per embryo were counted. (**D**, **E**) BrdU/Ki67 double label at E11.5. Note many BrdU+ and BrdU/Ki67 double-positive cells in mutant but not wild type RPE. (**F**) RT-PCR confirms the expression of FGF receptor-1 (*Fgfr1*) and 2 (*Fgfr2*) in both RPE and retinal domains. For details on tissue separation and RT-PCR conditions, see [8]. (**G**) Western blots for the indicated proteins in wild-type OV cultures of E10.5−11 embryos (n = 3) kept for 5 hours in DMEM serum-free medium in the presence or absence of human FGF2 and/or MEK1/2 inhibitor (MEKi) PD98059. Note reduction of p-ERK, p-H3, and CYCLIN D in presence of MEKi, regardless of whether FGF2 was added. (**H–J**; **N–P**) Mitotic cells (p-H3 positive) in *Mitf ^−/−^* (**H–J**) and *Vax2^−/−^; Mitf ^−/−^* (**N–P**) mutant RPE in OV cultures exposed for 36 hours to acrylic beads coated with FGF2, MEKi, or FGF2+MEKi. Note that these are horizontal sections and that placement of an FGF2 bead alone eventually leads to overgrowth of the entire RPE [7]. (**K–M**) VSX2 expression in *Mitf ^−/−^* mutant RPE in OV cultures exposed for 36 hours to acrylic beads coated with FGF2, or FGF2+MEKi. Note VSX2 expression and overgrowth in the vicinity of the FGF2 bead and inhibition of VSX2 expression and overgrowth in the vicinity of the FGF2/MEKi bead. Images shown are from 1 embryo each out of 5 per conditions giving similar results. Scale bar: 60 µm (**A**, **B**, **H–P**); 25 µm (**D**); 15 µm (**E**).
(TIF)

Figure S4 (A–F) VSX2/SOX2 and (G,H) JAGGED1 expression in wild-type and *Mitf* single or *Vax2/Mitf* double mutant eyes. Note SOX2 and VSX2 expression in the thickened RPE of *Vax2/Mitf* double mutants at E10.5 (**C**, arrow) and both *Mitf* single and *Vax2/Mitf* double mutants at later stages (**E,F**). Also note that the distal RPE remains largely free of SOX2 staining, marking it as ciliary margin RPE. Wild-type whole embryo cultures (n = 3 per condition) exposed for 36 hours to DMSO (**G**) or NOTCH antagonist γ-secretase inhibitor 1 (**H**). Note that JAGGED1 is expressed in the distal RPE domains in presence of γ-secretase inhibitor 1. Scale bar: 60 µm (**A–C**, **G**, **H**), 80 µm (**D–F**).
(TIF)

Table S1 (DOCX)
