# Supplementary figures and images for: Vax1/2 Genes Counteract Mitf-Induced Respecification of the Retinal Pigment Epithelium
Source: PLoS One. 2013 Mar 15;8(3):e59247. doi: 10.1371/journal.pone.0059247 (PMC3598659; doi:10.1371/journal.pone.0059247)

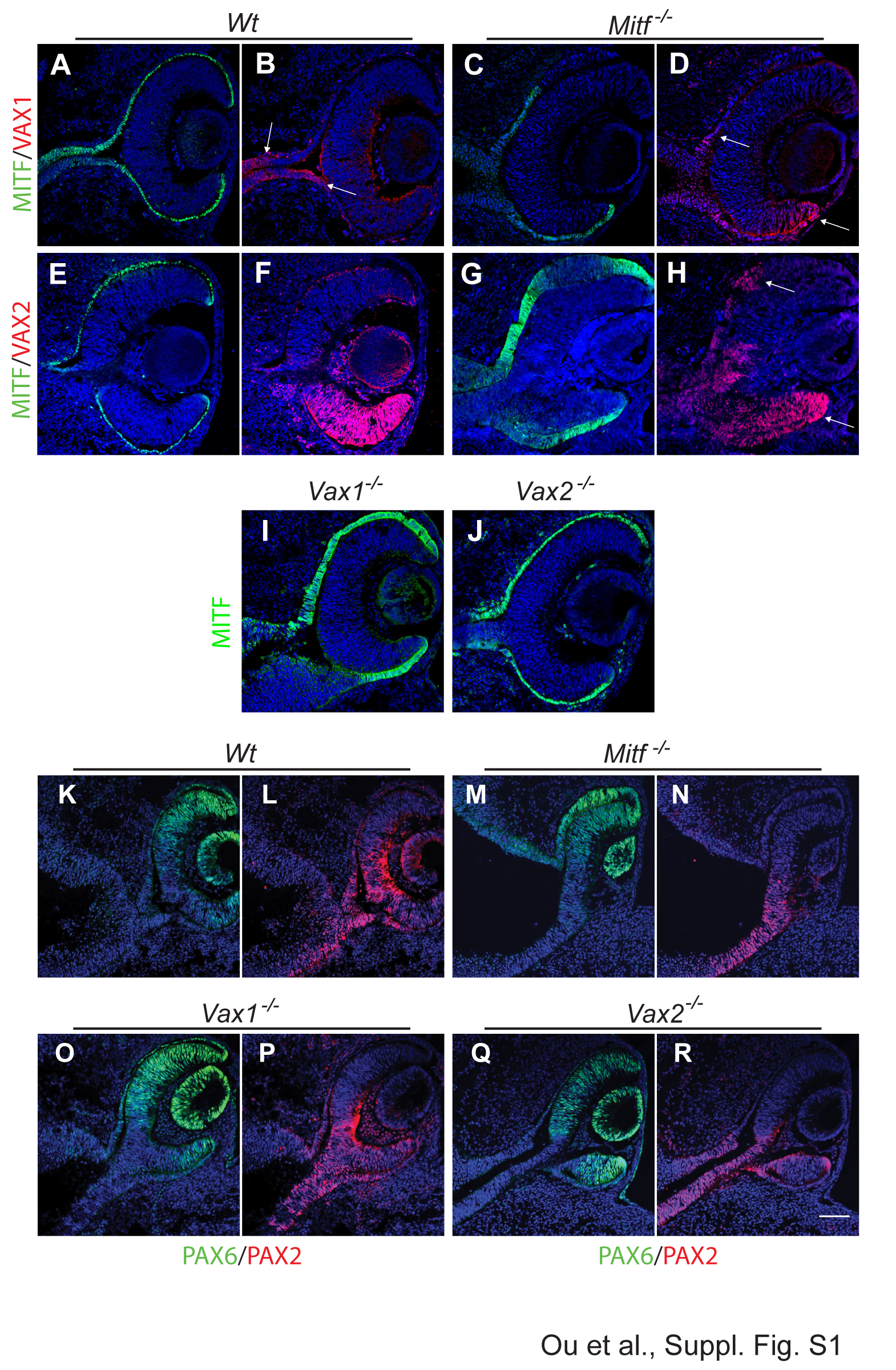

Supplement: Figure S1 — Expression patterns of MITF, PAX6 and PAX2 remain largely unchanged in Vax1, Vax2 and Mitf single mutant optic cups. Single channel confocal images of MITF and VAX1, and MITF and VAX2 expression patterns in E12.5 wild type (Wt; A,B,E,F), and Mitf −/− (C,D,G,H). Arrows indicate the expression of VAX proteins in the Wt optic stalk (B) and Mitf −/− RPE (D,H). MITF expression is normal in the RPE of Vax1−/− (I) and Vax2−/− mutants (J). (K–R) Normal expression of PAX6 and PAX2 in retina and OS of Wt and mutant embryos. Note enhanced PAX6 expression in the dorsal RPE of Mitf −/− mutants, confirming previous observations [19]. Scale bar: 80 µm. (TIF) [file pone.0059247.s001.tif]

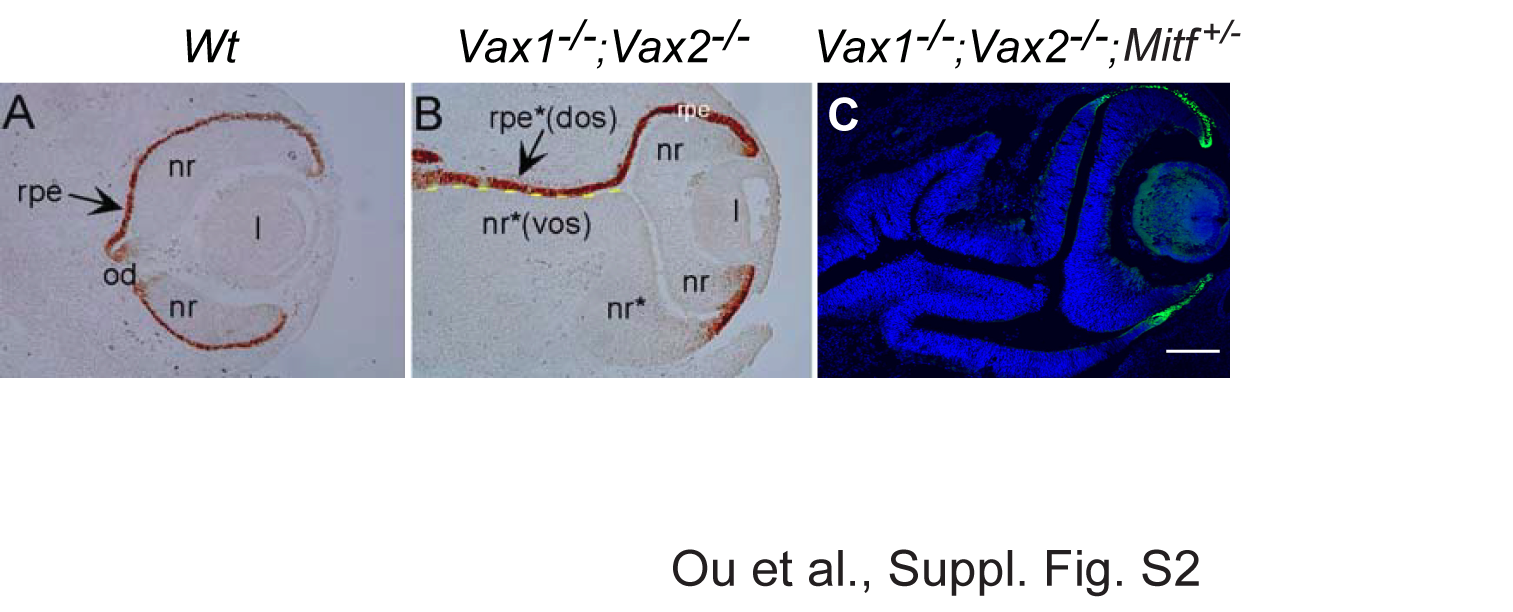

Supplement: Figure S2 — MITF expression in Vax1/Vax2 double mutants. Compared to wild type (A), MITF expression is expanded into the dorsal OS in Vax1/Vax2 double homozygous mutants at E14.5 (B). (C) Interestingly, Vax1−/−;Vax2−/−;Mitf +/− mutants show dorsal RPE thickening and loss of MITF expression in the dorso-proximal RPE but retention of MITF expression in the distal RPE. Scale bar: 180 µm. (TIF) [file pone.0059247.s002.tif]

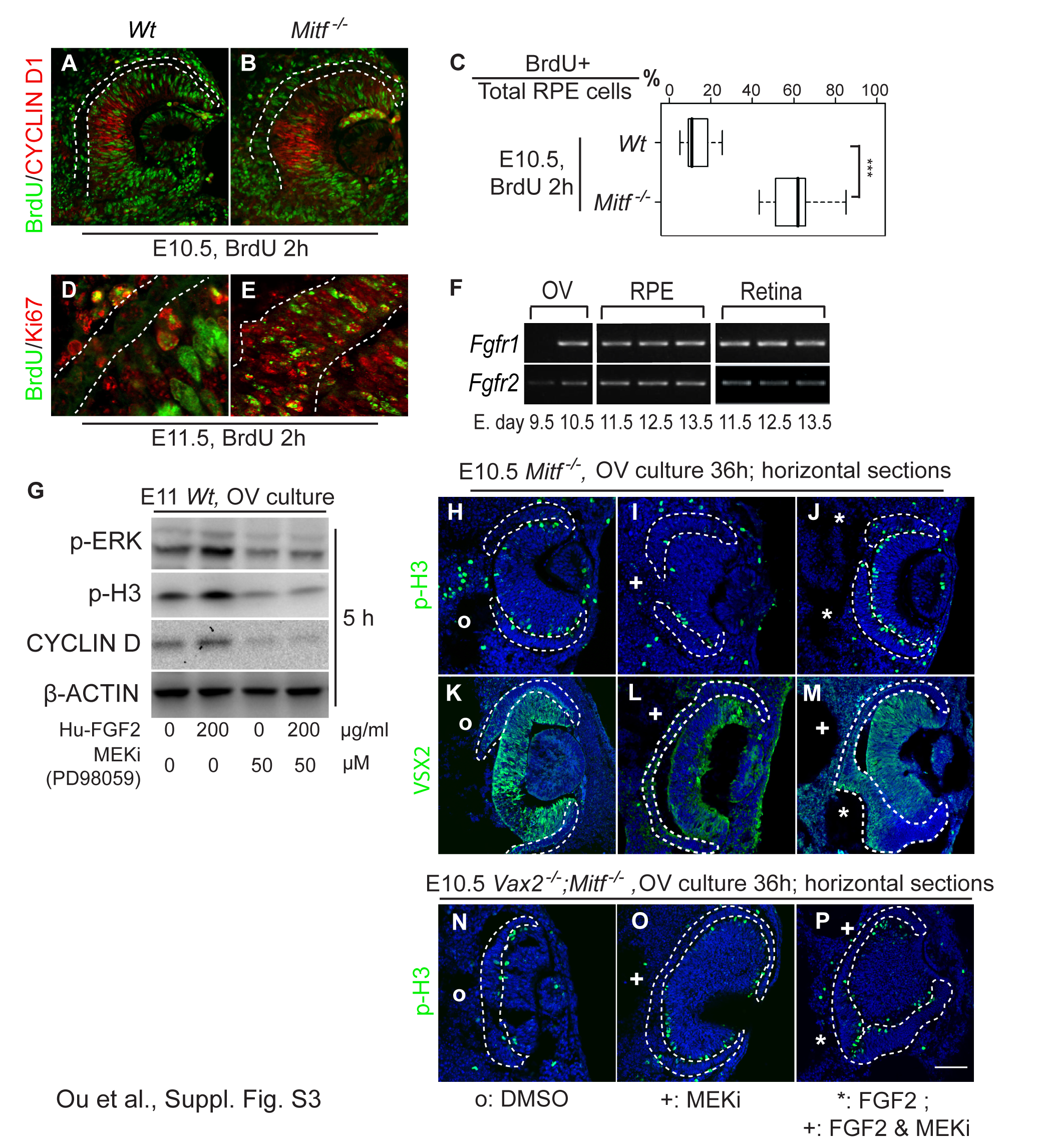

Supplement: Figure S3 — The thickening of Mitf mutant RPE is associated with cellular hyperproliferation. BrdU was injected intraperitoneally into pregnant mice, and mice were sacrificed 2 hours thereafter. Embryos were fixed and sectioned coronally. Sections were stained with antibodies against BrdU and double labeled for CYCLIN D1 or Ki67. (A, B) At E10.5, BrdU/CYCLIN D1 double label in wild-type and Mitf −/− eyes. Already at this stage before overt dorsal RPE thickening, Mitf −/− RPEs show increased BrdU labeling compared to wild type. (C) Quantitation of BrdU positive cells/per total cells in the dorsal RPE subdomain of wild type and Mitf −/− embryos. Box plots show minimal, 25th percentile, median, 75th percentile and maximal values of the respective percentages. Significance determined by Student’s t-test: ***: p<0.001. For quantitation, 6–10 embryos of each genotype and 2–3 sections per embryo were counted. (D, E) BrdU/Ki67 double label at E11.5. Note many BrdU+ and BrdU/Ki67 double-positive cells in mutant but not wild type RPE. (F) RT-PCR confirms the expression of FGF receptor-1 (Fgfr1) and 2 (Fgfr2) in both RPE and retinal domains. For details on tissue separation and RT-PCR conditions, see [8]. (G) Western blots for the indicated proteins in wild-type OV cultures of E10.5−11 embryos (n = 3) kept for 5 hours in DMEM serum-free medium in the presence or absence of human FGF2 and/or MEK1/2 inhibitor (MEKi) PD98059. Note reduction of p-ERK, p-H3, and CYCLIN D in presence of MEKi, regardless of whether FGF2 was added. (H–J; N–P) Mitotic cells (p-H3 positive) in Mitf −/− (H–J) and Vax2−/−; Mitf −/− (N–P) mutant RPE in OV cultures exposed for 36 hours to acrylic beads coated with FGF2, MEKi, or FGF2+MEKi. Note that these are horizontal sections and that placement of an FGF2 bead alone eventually leads to overgrowth of the entire RPE [7]. (K–M) VSX2 expression in Mitf −/− mutant RPE in OV cultures exposed for 36 hours to acrylic beads coated with FGF2, or FGF2+MEKi. Note VSX2 expressio [file pone.0059247.s003.tif]

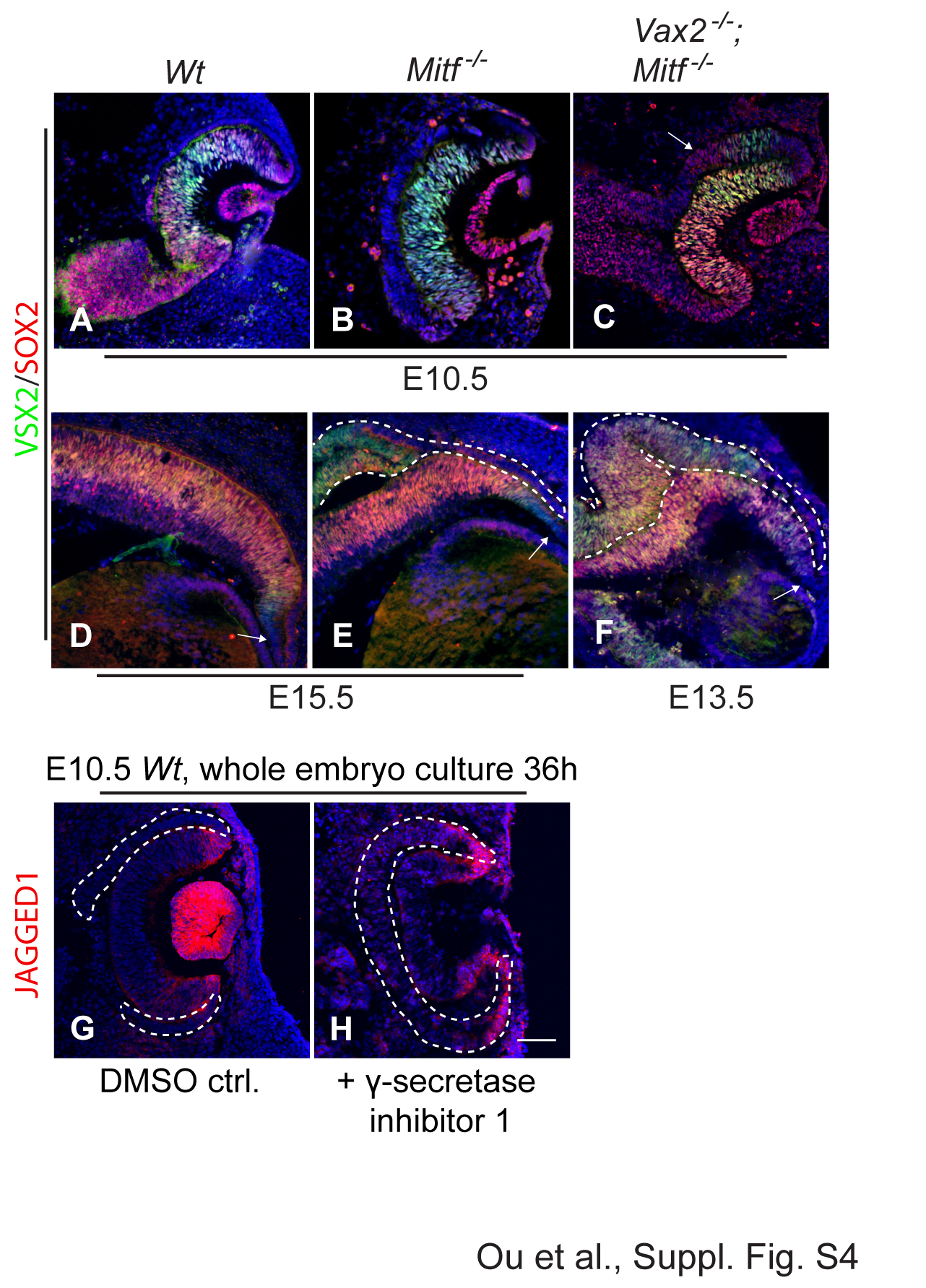

Supplement: Figure S4 — (A–F) VSX2/SOX2 and (G,H) JAGGED1 expression in wild-type and Mitf single or Vax2/Mitf double mutant eyes. Note SOX2 and VSX2 expression in the thickened RPE of Vax2/Mitf double mutants at E10.5 (C, arrow) and both Mitf single and Vax2/Mitf double mutants at later stages (E,F). Also note that the distal RPE remains largely free of SOX2 staining, marking it as ciliary margin RPE. Wild-type whole embryo cultures (n = 3 per condition) exposed for 36 hours to DMSO (G) or NOTCH antagonist γ-secretase inhibitor 1 (H). Note that JAGGED1 is expressed in the distal RPE domains in presence of γ-secretase inhibitor 1. Scale bar: 60 µm (A–C, G, H), 80 µm (D–F). (TIF) [file pone.0059247.s004.tif]
