# Supplementary material for: Vax1/2 Genes Counteract Mitf-Induced Respecification of the Retinal Pigment Epithelium
Source: PLoS One. 2013 Mar 15;8(3):e59247. doi: 10.1371/journal.pone.0059247 (PMC3598659; doi:10.1371/journal.pone.0059247)
Supplement: Table S1 — (DOCX) [file pone.0059247.s005.docx]

**Supplementary Table 1.**

| **#** | **Antibody** | **Species** | **Company** | | **Dilution** |
| --- | --- | --- | --- | --- | --- |
| 1 | anti-Mitf | mouse | Bharti et al, 2008 | | 1:1000 |
| 2 | anti-VAX1 (Bertuzzi et al., 1999) | rabbit | Gift from Dr. Greg Lemke (Salk Institute, La Jolla, CA, USA) | | 1:500 |
| 3 | anti-VAX2 (Kim et al., 2006) | rabbit | Gift from Dr. Greg Lemke (Salk Institute, La Jolla, CA, USA) | | 1:1000 |
| 4 | anti-Vsx2 | sheep | Exalpha Biologicals, (Shirley, MA, USA) | | 1:300 |
| 5 | anti-Vsx2 | rabbit | Gift from Dr. Connie Cepko (Harvard Medical School, Boston, MA, USA) | | 1:1000 |
| 6 | anti-Pax6 | rabbit | Covance (Princeton, NJ, USA) | | 1:500 |
| 7 | anti-Pax6 | mouse | [Developmental Studies Hybridoma Bank](http://dshb.biology.uiowa.edu/) (East Iowa city, IA, USA) | | 1:200 |
| 8 | anti-Pax2 | rabbit | Covance (Princeton, NJ, USA) | | 1:500 |
| 9 | anti-TUJ1 | mouse | Covance (Princeton, NJ, USA) | | 1:1000 |
| 10 | anti-Jagged1 | goat | Santa Cruz Biotechnology (Santa Cruz, CA, USA) | | 1:500 |
| 11 | anti-phospho-histone H3 | mouse | Cell Signaling Technologies ((Danvers, MA, USA) | | 1:500 |
| 12 | anti-phospho-Erk1/2 | rabbit | Cell Signaling Technologies (Danvers, MA, USA) | | 1:100 |
| 13 | anti-BrdU | mouse | BD Biosciences (San Jose, CA, USA) | | 1:500 |
| 14 | anti-cyclin D1 | mouse | Santa Cruz Biotechnology (Santa Cruz, CA, USA) | | 1:300 |
| 15 | anti-Sox2 | Rabbit | Millipore (Billerica, MA, USA) | | 1:500 |
| 16 | Anti-Ki67 | Rabbit | Abcam (Cambridge, MA, USA) | | 1:500 |
| 17 | Anti-Tyrosinase | Rabbit | Gift from Dr. Vincent Hearing, NCI | | 1:500 |
| 18 | anti-mouse IgG1-alexafluor 488 | goat | Invitrogen (Eugene, OR, USA) | | 1:800 |
| 19 | anti-goat IgG-alexafluor 488 | donkey | Invitrogen (Eugene, OR, USA) | | 1:800 |
| 20 | anti-sheep IgG-alexafluor 488 | donkey | Invitrogen (Eugene, OR, USA) | | 1:800 |
| 21 | anti-rabbit IgG-alexafluor 568 | donkey | Invitrogen (Eugene, OR, USA) | | 1:1000 |
| 22 | anti-mouse IgG2a-alexafluor 568 | goat | Invitrogen (Eugene, OR, USA) | | 1:1000 |
| 23 | anti-mouse IgG2a-alexafluor 633 | goat | Invitrogen (Eugene, OR, USA) | | 1:1000 |
| 24 | Normal rabbit IgG | rabbit | Santa Cruz Biotechnology (Santa Cruz, CA, USA) | | N/A |
| 25 | Normal goat IgG | goat | Santa Cruz Biotechnology (Santa Cruz, CA, USA) | | N/A |
|  | ***In situ* hybridization probe** | | **Company** | | |
|  | *Fgf15* | | Open Biosystems (Huntsville, Al, USA) | | |
| **#** | **Reverse transcription PCR primers** | | | **Amplification** | |
| 1 | *Fgfr1:* for: 5’ –GCTGGAAGTGCCTCCTCTTCTGG – 3’  rev: 5’ –CGCAGCCAGTTGATGCTCTGCAC– 3’ | | | 25 cycles | |
| 2 | *Fgfr2:* for: 5’ –TGGTCACCATGGCAACCTTGTCC– 3’  rev: 5’ –GAGATACTCCCCAATAAGCACTGTCC– 3’ | | | 25 cycles | |
